# Patient characteristics in German allergological practices – a nationwide survey 

**DOI:** 10.5414/ALX01348E

**Published:** 2018-09-01

**Authors:** T. Reinhold, C. Lindig, S.N. Willich, B. Brüggenjürgen

**Affiliations:** Institute for Social Medicine, Epidemiology and Health Economics, Charité – University Medical Center, Berlin, Germany

**Keywords:** specific immunotherapy, patient characteristics, cross-sectional survey, sublingual immunotherapy, subcutaneous immunotherapy

## Abstract

Introduction: In Western societies a significant incidence and prevalence of allergic asthma and other allergic diseases is observable. The present study investigated epidemiological patterns of allergic diseases and the utilization of health care resources by subjects who are already under specialized allergological treatment. Furthermore the study was performed to identify factors which had a significant impact on accessibility to specific immunotherapy (SIT). Methods: The study was based on a cross-sectional survey on patient characteristics, which was performed by participating physicians, who were specialized in the field of allergological disorders and SIT, in collaboration with their patients. The analysis of data was divided into descriptive analyses and an analytical part, in which influencing factors for accessibility to specific immunotherapy were investigated. Logistic regression models to identify several predictor variables were used. Results: 495 physicians documented the data of 19,990 patients. 18,177 patients were included in the analyses. Patients had a mean age of 31.5 ± 15.5 years and 53.2% were female. The most frequent and most severe allergic disorders observable in German allergological practices were conjunctivitis and rhinitis. The seasonal symptoms occurred mainly during March to August, while seasonal disease manifestation was 2.5 times more frequent than perennial forms. The most received anti-symptomatic medications are antihistamines and corticosteroids. Patients who receive SIT were mainly treated using subcutaneous immunotherapy (SCIT) – only in lower age groups, the likelihood of receiving sublingual immunotherapy (SLIT) was increased. Conclusion: In Germany, conjunctivitis and rhinitis are the most severe allergic disorders in allergological practices. Compared to the German general patient population, people who were already in allergological treatment had better access to SIT.

**German version published in Allergologie, Vol. 34, No. 5/2011, pp. 237-247**

## Introduction 

In Western societies a significant incidence and prevalence of allergic asthma and other allergic diseases is observable. The frequency of these disorders has increased alarmingly during the past three decades and is still growing in industrialized countries as well as in the developing world [1, 2]. Therefore allergic diseases constitute a growing health problem in relation to morbidity [3]. 

About 25% of people living a Western lifestyle suffer from allergic rhinitis [4]. It constitutes one of the leading causes for significant morbidity, including quality of sleep, headache, cognitive impairment and other systematic symptoms, as well as affecting social life and productive efficiency [5, 6]. For evaluating treatment against allergic rhinitis, the correlation of allergic rhinitis and allergic asthma should be taken into consideration. A number of studies have suggested that children who suffer from allergic rhinitis are three times more likely to develop adult-onset asthma [7, 8]. Results of other investigations indicate that about 80% of patients with allergic asthma also suffer from symptoms of allergic rhinitis [9]. 

Recent studies showed no clear differences in future trends of asthma prevalence with regard to disease severity or age. Additionally, no differences were found between developed and developing countries [10]. Some studies are based on actual diseases diagnosed by physicians while other studies allude to symptoms according to patients statements. It seems that the prevalence of symptoms associated with asthma or other allergic diseases underlie greater variation than the prevalence of diagnosed diseases [10]. Thus the worldwide prevalence of asthma symptoms, symptoms of allergic rhinoconjunctivitis, and symptoms of eczema show large variations. Due to the fact that these variations can even be found in genetically homogeneous populations, environmental factors seem to have an important impact on the frequency of symptoms [11, 12, 13]. The prevalence of asthma ranges between about 2% in Albania or Russia and about 20% in Australia and New Zealand. The data of rhinoconjunctivitis range between 5% in the Baltic States and over 30% in countries such as Malta, Paraguay or Nigeria [11]. In Germany the prevalence of asthma is at 3% to 5% in adults and around 10% in children [14, 15]. Total asthma prevalence is projected to be around 6% (6.34%) [15]. 

The increasing prevalence of allergic diseases is also associated with an increase in health care costs [16, 17, 18, 19]. Economical aspects have an impact on treatment choices and health status. Thus physicians have to identify the most efficient solutions to improve their patients health status while not abstracting away from health care costs in order to consider the cost-effectiveness ratio of every medical treatment [16]. The economic burden of allergic diseases in Germany, containing the direct costs derived from the number of physician visits, drug treatment, and the indirect costs related to reduced productivity are estimated at EUR 3.5 billion during the late 1990s [20]. Consequently, any preventive policy aimed at reducing the impact of allergic diseases, such as the clinical treatment of severe complications, can reduce health care costs [21]. 

Many patients have only mild symptoms that are sometimes ignored, sometimes treated with antihistamine tablets [22]. Depending on the severity of symptoms, corticosteroid sprays are applied additionally [22]. Although pharmacotherapy can alleviate symptoms in many cases, in others they are hard to control and require allergen-specific immunotherapy [23, 24]. Specific immunotherapy (SIT) is able to influence the course of allergic disease and is currently the only causal therapy of allergic diseases and routinely performed modulation of the immune system [6, 25, 26, 27]. Great efforts are made continuously to improve SIT and reduce side effects. Technological advances in the field of production of recombinant allergens ensure a high standard of clinical use [28, 29]. Beside the most commonly used subcutaneous immunotherapy (SCIT), sublingual immunotherapy (SLIT) is now a treatment option which is used with increasingly frequency both in Europe and the USA [29, 30, 31, 32]. 

Currently, economic evaluations of this treatment are rare, particularly in a German context. Using a retrospective 10-year decision model, Buechner et al. detected a 50% reduction of direct and indirect costs in patients with allergic rhinitis and asthma treated with SIT compared with those treated with symptomatic drugs [33]. A recently published study on cost-effectiveness of specific SCIT found annual cost savings of approximately EUR 140,– per SCIT-treated patient from societys perspective [34]. 

## Objective 

The main objectives of the present study were to get more information on

- epidemiological pattern of allergic diseases (sensitization pattern, range of symptoms, frequency of symptoms and symptom interdependence) and the
- utilization of medication by subjects suffering from allergic diseases and patients who are already under specialized allergological treatment.

A secondary aim was to identify factors which had a significant impact on access to SIT. 

## Methods 

### Study setting 

The study was based on a cross-sectional survey which was performed in Germany between July 2005 and December 2007. Physicians were asked to participate if they were specialized in the field of allergological disorders and the emphasis of their daily work was also on SIT (> 50 SIT per year). If they were eligible and willing to participate, they were asked to document characteristics of their allergy-patients during a doctors-visit. This documentation was to be produced in cooperation with the patients, particularly in measuring the individual medical condition. 

The documentation was performed using standardized questionnaires, which were structured into different parts:

- Socioeconomic patient data,
- data on allergic disorders before any treatment onset (including symptom scores from 1 to 10),
- kind of any symptomatic therapy approaches,
- data on previous diagnostic procedures, and
- information on the use of specific immunotherapy.

After the documented patients gave their informed consent, the data acquisition was done in an anonymized way. Subsequent to the data collection and independently from each other, two researchers checked all documented data for plausibility and informative value. If this was not ensured, these questionable data were excluded from subsequent analyses. Differences in plausibility appraisal between the researchers were resolved in discussions. 

### Statistical analyses 

The analysis of data was divided into descriptive analyses as well as an additional analytical part, in which influencing factors for access to specific immunotherapy were investigated. For this purpose, we used a binary logistic regression analysis to identify several predictor variables. To receive immunotherapy (yes/no) was defined as a dependent variable. Beside categorical variables like gender, health insurance affiliation (statutory health insurance/private health insurance), kind of symptomatic medication (antihistamines yes/no, mast cell-stabilizing agents yes/no, corticosteroids yes/no, betamimetics yes/no, theophyllines yes/no, others yes/no), we also considered continuous variables like age or intensity of allergic conditions (symptom score) as potential predictor variables. Additionally we investigated some specific subgroups with respect to, maybe unexpected, differences. 

If missing data were found, these subjects were excluded from specific analysis without using any methods for replacement of missing data. 

The association between receiving immunotherapy and potential impact variables is described using odds ratios (OR) and 95% confidence intervals (95% CI). Statistical significance was defined by a 2-sided alpha level of 0.05. Statistical analyses were performed using PASW statistics 18 version 18.0.0. 

## Results 

### Baseline characteristics 

**Figure 1.**
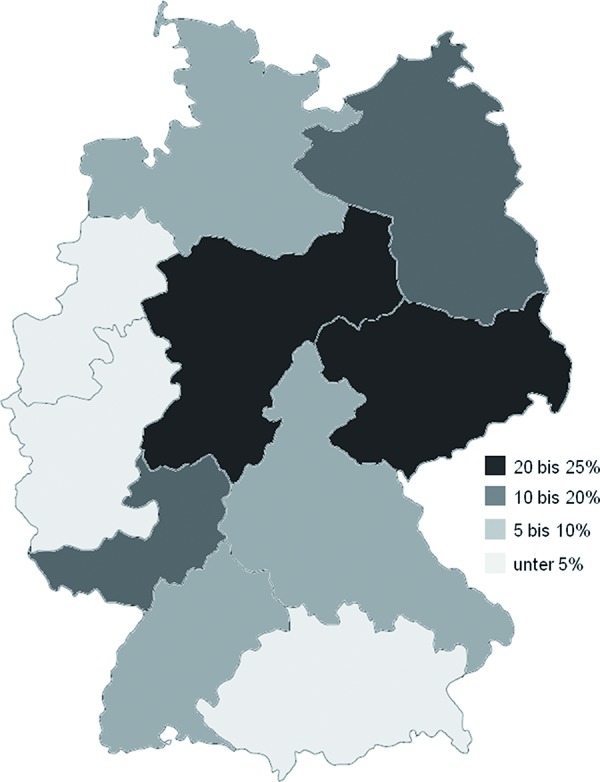
Local allocation of documented patient data across Germany in percent of all included patients (based on postal code are as documented on filled questionnaires).

Of the 597 doctors invited initially, 495 physicians participated in our study. Participating doctors were mainly specialists in otolaryngology (41%), followed by dermatologists (36%) and pneumologists (13%), pediatricians (5%) and internists/general practitioners (each 3%). These physicians documented the data of 19,990 patients. After checking for plausibility and informative value, we excluded the data of 1,813 patients. The most frequent reasons for excluding data were missing information on patients age, no documented information on allergic disorders as well as missing information regarding the duration of allergic symptoms. Finally, the results are based on a total number of 18,177 patients. These patients had a mean age of 31.5 ± 15.5 years. The proportion of female patients was 53.2%. Most of the documented patients (86.7%) were members of German statutory health insurance services. This corresponds to the insurance status of the German total population where the proportion of compulsory insured persons was 87.8% [35]. An overview on the main characteristics of documented patients is given in Table 1. The recruitment of patients was focused on central Germany, so the allocation of documented patients does not represent the real geographical population distribution in Germany (Figure 1). 

### Allergic suffering patterns 

**Figure 2.**
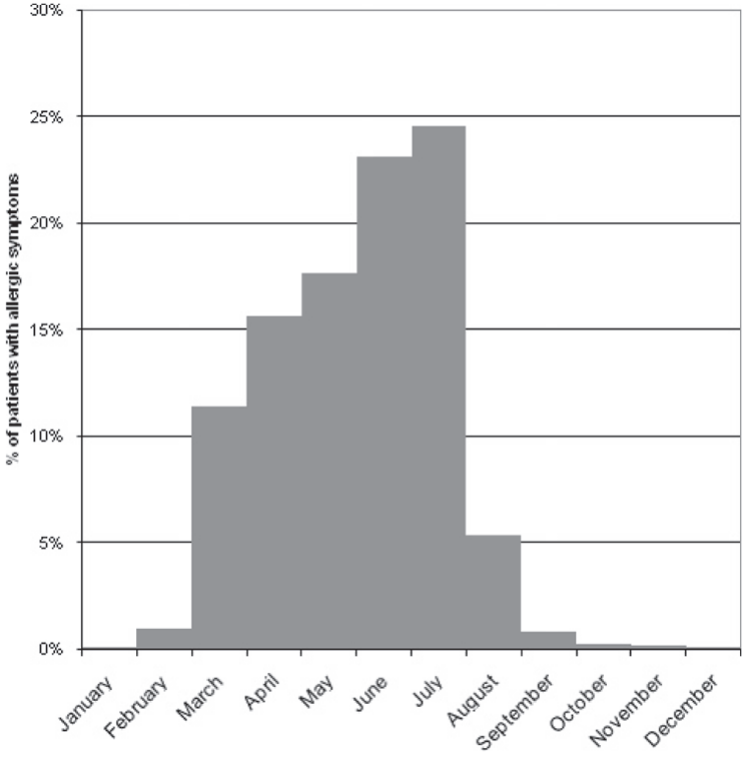
Proportion of patients suffering from allergic symptoms and seasonal dependency.

**Figure 3.**
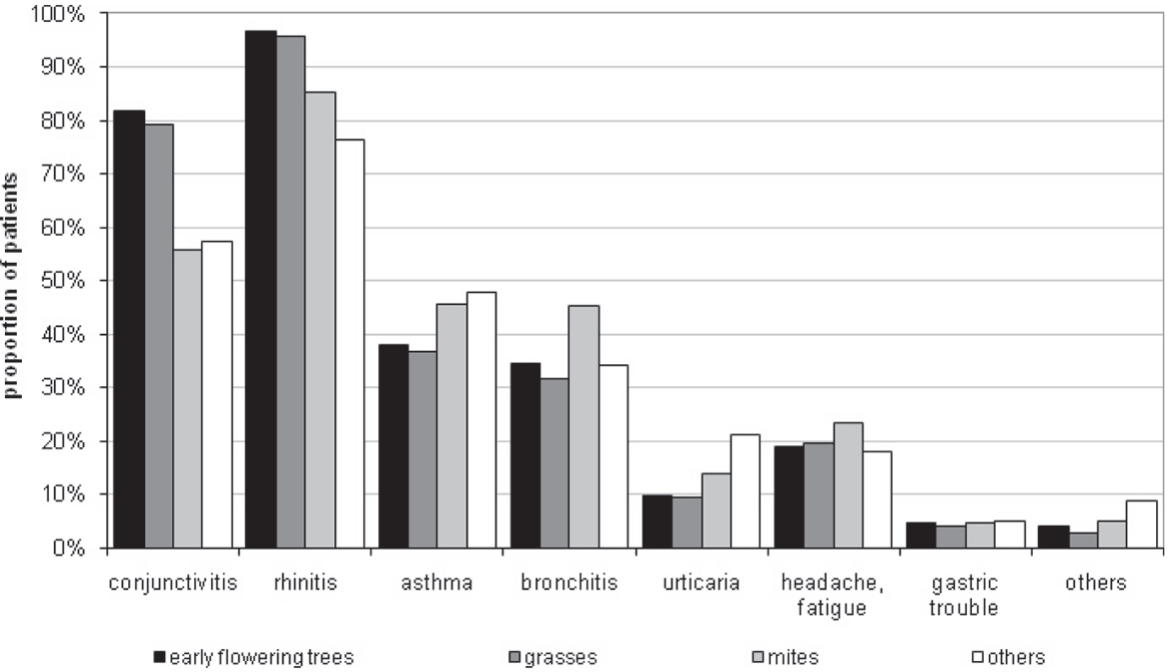
Frequency of clinically manifested sensitizations by type of allergen.

The most frequent allergens responsible for allergic disorders were early flowering trees and grasses, each with around 50% of all documented patients. 27% of patients suffer further from hypersensitivity to mites (multiple answers possible). Other allergens were observed to be more rare (e.g. hypersensitivity to mugwort, cat, moulds). The proportion of patients with perennial allergic symptoms was 94% for mites and 60% for the category “other allergens”. Overall, the proportion of perennial vs. seasonal disease manifestation was 1: 2.5. Of those patients who were not affected by perennial types of allergy, the allergic symptoms reach the peak of frequency during July, when 25% of all investigated patients are affected. During September to February the overall frequency of allergy symptoms is the lowest (Figure 2). Nearly all documented patients suffered from allergic rhinitis, followed by conjunctivitis, asthma and bronchitis, headache/fatigue and urticaria (Figure 3). We observed marked differences in the frequency of allergic disorders according to the type of allergens. While rhinitis in about 95% and conjunctivitis in approximately 80% of all patients are mostly associated with a hypersensitivity to early flowering trees and grasses, asthma and bronchitis were more common in patients who are hypersensitive to mites. Measured by their prevalence, other allergic disorders such as gastric trouble seem to be negligible. 

**Figure 4.**
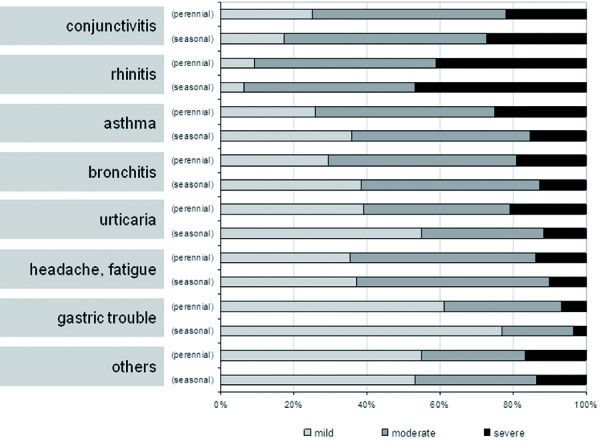
Allergic disorders and the related severity of symptoms.

Beside the frequency of allergic disorders, we were also interested in obtaining information on the intensity of existing medical conditions. As shown in Figure 4, for many patients allergic rhinitis is associated with moderate or severe symptoms. Interestingly, symptoms of rhinitis as well as conjunctivitis were felt as more severe in seasonal forms compared to perennial manifestation. All other allergic disorders were assessed as less severe in the case of seasonal manifestation. 

**Figure 5.**
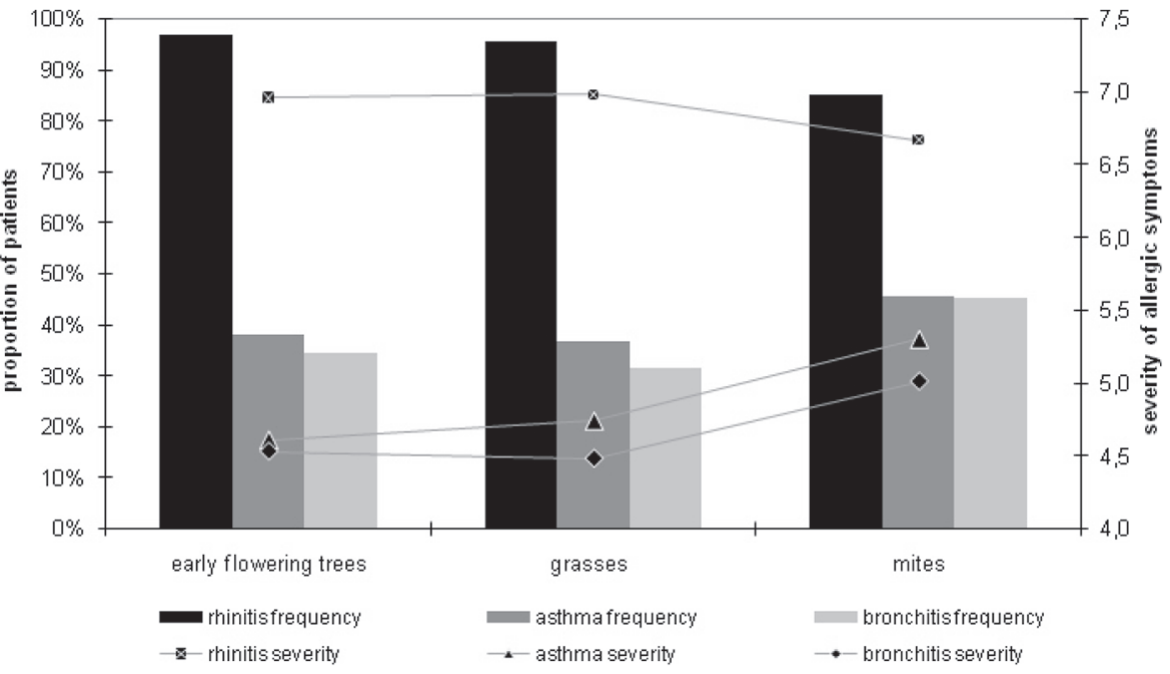
Disease frequency and severity by type of allergen.

An additional view on self-reported intensity of symptoms divided by type of allergens leads to another clear conclusion: in patients who were hypersensitive to mites, the frequency as well as the intensity of allergic rhinitis is lower than in patients who are allergic to early flowering trees or grasses. Another picture may be observed when looking at allergic asthma and bronchitis. As summarized in Figure 5, allergic asthma and bronchitis were not only associated with higher prevalence in patients allergic to mites, but also with more severe symptoms as shown by its ranking in the symptom severity score. 

### Anti-symptomatic medication patterns 

**Figure 6.**
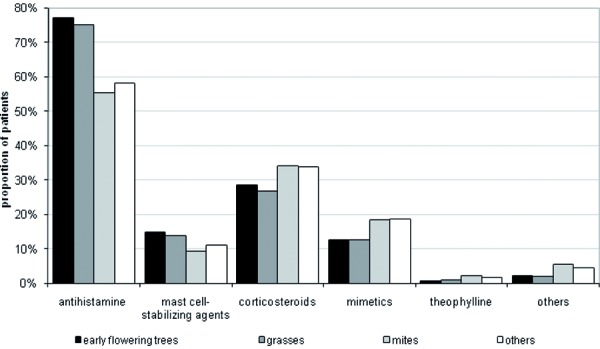
Use of symptomatic medication by type of allergen.

Another topic of interest was the physicians behaviour regarding the prescription of anti-symptomatic drugs. Figure 6 shows that antihistamines were the most common medication regardless of the underlying reason for the allergic disorder – at least every second patient was an antihistamine user. A more detailed view shows that there are large differences associated with the type of allergen. For instance, corticosteroids were used more frequently in patients with intolerance to mites (~ 34%) compared to patients with hypersensitivity to early flowering trees or grasses (~ 27%). Observing antihistamine usage in more detail, the opposite situation is detectable (~ 55% vs. ~ 75%). 

### Specific immunotherapy utilization patterns 

**Figure 7.**
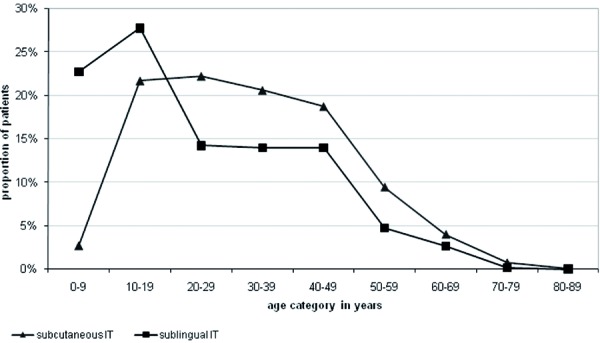
Age distribution in relation to type SIT application.

Of the 18,177 patients involved initially in participating allergological practices, 15,052 persons (82.8%) received SIT to improve their condition. SIT patients had a slightly lower age of 31.1 ± 15.1 years compared to patients without SIT (33.1 ± 17.2 years). Patients receiving SIT also suffer from a higher overall symptom score. A detailed analysis of single disease symptoms is provided in our regression analyses. In those patients who were treated with SIT, a majority received perennial therapy (approximately 65%) instead of preseasonal approaches. The type of application is dominated by subcutaneous intake (SCIT) that was preferred to sublingual therapy (SLIT) in 95% of all cases. SLIT is more frequently applied to younger patients – so 20% of all SLIT patients received the treatment in age category from 0 to 9 years vs. only 3% in subcutaneous application. Similar differences were observable in age category from 10 to 19 years (Figure 7). In advanced age-groups the reverse situation emerged. 

Using regression analyses we tried to identify influencing factors for receiving SIT. As mentioned in Table 2, the most favorable factors were younger age, severe complaints due to conjunctivitis, rhinitis and asthma (each p ≤ 0.001). Patients with mild symptoms of urticaria, headache/fatigue and gastric troubles are more likely to receive SIT compared to patients with related severe complaints. 

## Discussion 

The present study gives an overview of important patients-characteristics in German allergological practices. The main results can be summarized as follows:

- The most frequent as well as most severe allergic disorders observable in German allergological practices were conjunctivitis and rhinitis.
- Seasonal symptoms occurred mainly during the period from March to August.
- Seasonal disease manifestation was 2.5 times more frequent compared to perennial forms.
- The most received anti-symptomatic medications are antihistamines and corticosteroids.
- When patients receive SIT, they were mainly treated using SCIT – only in lower age groups, did the likelihood of receiving SLIT increase.

Some of our main results were strongly supported by further investigations and will confirm usual hypotheses on the development of allergic diseases. For instance, regarding the allergic suffering patterns we found, that pollen-driven allergic disorders were more frequent than mites or other allergens. This was also observed within the telephone health survey performed by the German Robert Koch Institute. They also reported pollen allergens almost twice as frequently as mites (21.1% vs. 13.6%) [36]. The higher overall frequency of pollen-hypersensitivity of 50% we found in our study is explained by the selected patients of our study, while the telephone survey was targeted at the general population. Another prospective observational study which focused on patients of allergological practices came to similar conclusions as we did in our analysis [37]. Indeed this study reported a lower rate of mite-hypersensitivity than we found in our investigation (approximately 10 – 12% vs. 27%), but this correlates with a higher proportion of asthma frequency of approximately 40% in the present analysis compared to 27% investigated by Thum-Oltmer et al. 2005. So the assumption that asthma is more common in patients with existing hypersensitivity to mites, is confirmed again [38]. Further findings presented in our study, for example on symptomatic medication usage or the specific immunotherapy utilization patterns, were not supported by further analysis due to a lack of German studies suitable for comparison. 

To our knowledge, the present paper is the first investigation with focus on patient characteristics in German allergological practices using such large numbers of respondents. Beside this significant strength of our study, readers have to keep some potential limitations in mind that could influence the interpretation of results. In terms of insurance status and gender distribution the investigated population is representative compared to the German norm population. Nevertheless, the survey was performed in a selected setting, so selection bias is likely. For instance this could be seen with regard to access to SIT which was very high in our survey (around 80%) compared to studies investigating the German allergological norm-patient (around 10% [39]). 

Beside this quantitative under-supply in the general patient population it could be assumed that there is an additional qualitative under-supply with regard to physicians qualification, standardized treatment or documentation [40]. The main reason for this difference between our study population and findings in the general patient population is the fact that our patient sample was already in specialized allergological treatment. Thus, the barrier to receiving SIT is not as high as it is in patients without a previous contact to medical specialists. Additionally the sample of our study did not cover the German regions according to the size of population living there. That is also a reason for limited transferability of our findings to the German patient population as a whole. Some allergens or attendant parameters (e.g. local micro climate or local vegetation) may be different in quantity or quality compared to other, and within this survey, under-represented German regions. 

Another limitation lies in the nature of retrospective questions, as they were also used in our questionnaires. Such questions are characterized by their risk of recall bias. Recall bias represents a major threat to the internal validity of studies using self-reported data and is a classic form of information bias [41]. To get information on the dimension of medical complaints before an immunotherapy was started for example, patients were asked to report their condition independent of their actual treatment status. But if a patient already receives SIT, the question about his complaints before immunotherapy has a retrospective character and is therefore afflicted with the risk of recall bias. 

Last but not least, readers have to consider the risk of confounding. While bias involves error in the measurement of a variable, confounding involves error in the interpretation of what may be an accurate measurement. The consequence of this is that the estimated association is not the same as true effect. For example, in our study we found a positive association between utilization of SIT and younger age. At least in parts, these results could be influenced by confounding factors. Perhaps younger patients who contacted an allergologist suffer more severely from allergic disorders compared to patients who contacted these medical specialists 20 life-years later. Comparable confounding risks may be assumed with regard to the membership in German statutory health insurance that was also identified as a significant influence coefficient for SIT demands. 

## Conclusion 

In Germany, the most severe allergic disorders in observable allergological practices were conjunctivitis and rhinitis. The most frequent allergic suffering occurs during March to August. The patients treated their symptoms mainly using anti-symptomatic drugs such as antihistamines and corticosteroids. Compared to the German general patient population, people who were already in allergological treatment had better access to SIT. Additionally we detected age-specific differences in the proportion of subcutaneous vs. sublingual treatment approaches, with a wider use of sublingual immunotherapy in younger age-groups. 

## Conflicts of interest 

The survey was designed and performed by Allergopharma Joachim Ganzer KG, the data evaluation was made by the Institute for Social Medicine, Epidemiology and Health Economics at the Charité – University Medical Center, Berlin, Germany. The researchers received a non-restrictive research grant. 

**Table 1.** Socioeconomic key data of documented patients.

	all patients n = 18,177			
age in years (mean ± SD)	31.5 ± 15.5			
gender	female	male		
n (%)	9,662 (53.2%)	8,515 (46.8%)		
age in years (mean ± SD)	32.4 ± 15.2	30.4 ± 15.7		
insurance status	statutory health insurance	privately insured	statutory health insurance	privately insured
n (%)	8,691 (90.0%)	971 (10.0%)	7,074 (83.1%)	1,441 (16.9%)
age in years (mean ± SD)	33.4 ± 15.3	32.2 ± 14.7	29.7 ± 15.8	33.7 ± 14.9

**Table 2.** Results of regression analysis (n = 18,177).

	Regression coefficient	SE	p-value	OR	OR (95%-CI)		Interpretation (significant favorable factors for the use of SIT)
Sociodemographic variables							
Age	–0.007	0.001	≤ 0.001	0.993	0.991	0.996	Younger age
Gender	–0.044	0.041	0.288	0.957	0.882	1.038	–
n male: 8,515							
n female: 9,662							
Insurance status	0.174	0.059	0.003	1.191	1.061	1.336	Statutory health insurance member
n SHI: 15,765							
n PHI: 2,412							
Variables on allergic suffering							
Conjunctivitis n = 13,725	0.061	0.007	≤ 0.001	1.063	1.048	1.078	Severe complaints due to conjunctivitis
Rhinitis n = 17,038	0.182	0.008	≤ 0.001	1.199	1.181	1.218	Severe complaints due to rhinitis
Asthma n = 7,896	0.091	0.009	≤ 0.001	1.095	1.076	1.114	Severe complaints due to asthma
Bronchitis n = 7,237	0.000	0.008	0.993	1.000	0.984	1.016	–
Urticaria n = 2,411	–0.034	0.011	0.003	0.967	0.945	0.989	Mild complaints due to urticaria
Headache, fatigue n = 4,160	–0.027	0.010	0.007	0.973	0.954	0.993	Mild complaints due to headache/fatigue
Gastric symptoms n = 1,077	–0.107	0.022	≤ 0.001	0.899	0.860	0.938	Mild complaints due to gastric symptoms
Others n = 891	0.071	0.021	0.001	1.074	1.031	1.118	Severe complaints due to other allergic disorders
Variables on symptomatic medication							
Antihistamines n = 13,033	–0.061	0.047	0.189	0.941	0.859	1.031	–
Mast cell-stabilizing agents n = 2,663	–0.076	0.060	0.207	0.927	0.824	1.043	–
Corticosteroids n = 6,122	–0.004	0.048	0.932	0.996	0.907	1.094	–
Betamimetics n = 2,981	0.230	0.067	0.001	1.258	1.104	1.434	High utilization of betamimetics
Theophyllines n = 317	–0.620	0.140	≤ 0.001	0.538	0.409	0.708	Low utilization of theophyllines
Others n = 653	–0.081	0.106	0.446	0.922	0.748	1.136	–

SHI: statutory health insurance; PHI: private health insurance.
